# Validation of the Perceived Barriers to Antiretroviral Therapy Adherence (PEDIA) Scale Among Gay, Bisexual, and Other Men Who Have Sex With Men and Transgender and Nonbinary Persons: Cross-Sectional Study

**DOI:** 10.2196/67005

**Published:** 2025-06-27

**Authors:** Victor C Matos, Dyego Carlos Araújo, Thiago S Torres, Paula M Luz

**Affiliations:** 1 Escola Nacional de Saúde Pública Sergio Arouca, Fundação Oswaldo Cruz, Rua Leopoldo Bulhões, 1480, Rio de Janeiro, 21041-210, Brazil, 55 2138659623; 2 Departamento de Ciências Farmacêuticas, Universidade Federal do Espírito Santo, Vitória, Brazil; 3 Instituto Nacional de Infectologia Evandro Chagas, Fundação Oswaldo Cruz, Rio de Janeiro, Brazil

**Keywords:** antiretroviral therapy, adherence, perceived barriers, psychometric analysis, human immunodeficiency virus, Brazil, HIV

## Abstract

**Background:**

Approximately 30% of people living with HIV in Brazil have suboptimal adherence to antiretroviral therapy (ART). Theoretical models of health behaviors and objective evidence support the role of perceived barriers in hindering health behaviors.

**Objective:**

We aimed to evaluate the psychometric properties of the perceived barriers to ART adherence (PEDIA) scale among gay, bisexual, and other men who have sex with men (MSM) and transgender and nonbinary (TGNB) persons in Brazil.

**Methods:**

Using a cross-sectional study design, we evaluated the factor structure, construct validity, convergent and discriminant factorial validity, and internal reliability of the PEDIA scale using 2 convenience samples of participants aged 18 years or older living in Brazil. Sample 2020 was recruited through an internet-based survey between February and March 2020 and Sample 2021 between May 2021 and January 2022. The study instrument included sociodemographic information, ART use, and the 3 measures of ART adherence. Using Sample 2020, we performed exploratory factor analysis (EFA) with parallel analysis to evaluate PEDIA’s factor structure. Based on the goodness of fit measures and theoretical relevance, we developed a reduced version of PEDIA (PEDIAr). Using Sample 2021, we performed a psychometric evaluation of PEDIAr, which included confirmatory factor analysis (CFA), examination of modification indexes and item-item and item-total correlations, and an assessment of its correlation with different measures of adherence to ART. Goodness of fit was evaluated based on multiple indices.

**Results:**

EFA conducted on Sample 2020 (n=1692) revealed a 2-factor structure with 3 factor loadings <0.4 (excluded). Using Sample 2021 (n=4893), modification indices from the CFA and item-item and item-total correlations along with item relevance analysis suggested the exclusion of 5 additional items. CFA fit indices for PEDIAr were adequate (root mean square error of approximation=0.07, comparative fit index=0.95, Tucker-Lewis Index=0.94, standardized root mean square residual=0.05). Construct validity was supported by factor loadings above 0.6 and negative correlations between PEDIAr scores and 3 measures of adherence to ART. McDonald omega was 0.795 and 0.859 for factors 1 and 2, respectively. The square root of the average variance extracted (AVE) was 0.704 and 0.711 for factors 1 and 2, respectively, and the difference between AVE and the square of the factor correlations with other items was small (0.001 and 0.009, respectively) and not statistically significant for both factors (*P*=.94 and *P*=.55, respectively).

**Conclusions:**

PEDIAr, the 10-item reduced version of PEDIA, proved to be valid among gay, bisexual, and other MSM and TGNB persons in Brazil. This shorter instrument was able to capture 2 distinguished dimensions of the perceived barriers to adherence to ART (practical aspects and psychological aspects). By proactively identifying individuals struggling with adherence to ART, PEDIAr can facilitate timely interventions and improve personalized care.

## Introduction

Worldwide estimates point to 30.7 million out of 39.9 million (76.9%) people living with HIV with access to antiretroviral therapy (ART) at the end of 2023 [1]. However, ART adherence is a challenge for people living with HIV across multiple settings and populations [2]. Brazil’s HIV epidemic is characterized as a concentrated epidemic, with specific populations bearing a disproportionate burden of the disease. Among these groups, gay, bisexual, other men who have sex with men (MSM), and transgender and nonbinary (TGNB) persons are one of the most severely affected population groups. This population experiences significantly higher HIV prevalence [3] and HIV incidence rates [4]. In Brazil, nationally representative data on the cascade of HIV care is still scarce, with a 2016 study conducted in the city of Rio de Janeiro among 793 MSM and 37 transwomen indicating that 72.5% of those diagnosed were linked to care, 68.7% retained in care, and 61.1% were taking ART [5]. In terms of ART adherence in Brazil, a systematic review with meta-analysis that included 24 studies using various adherence measures published between 2005 and 2016 estimated that only 64% of people living with HIV achieved optimal ART adherence, frequently defined as ≥95% though it varied by study [6].

Numerous obstacles to achieve and sustain ART adherence have been investigated, notably at the individual level age, education, knowledge about ART, mental and physical health, and use of alcohol and illicit drugs [7-9]. Nonetheless, interpersonal factors have gained considerable attention, such as HIV-related stigma, lack of family and community support, and patient–health care provider mistrust [10,11]. In consonance, perceived barriers of people living with HIV, which denote an individual’s assessment of social, personal, and environmental hurdles to fulfill a goal [12], have risen in importance [13]. Evidence for other chronic diseases, such as cancer and heart failure, show that perceived barriers are strong predictors of health behaviors [14,15]. In the context of HIV care, perceived barriers of treatment refer to the struggles people living with HIV face to initiate and sustain ART adherence [16]. Daily ART adherence is the best predictor of viral suppression [17], and it hinders HIV transmission, disease progression, and the development of drug-resistant viral strains [18].

Person-reported outcome measures provide a unique perspective on health-related outcomes that are centered on the person as opposed to what is perceived by health professionals [19]. Existing person-reported instruments related to ART adherence focus on constructs such as HIV-related stigma [20], depression [21], and alcohol use [22]. These instruments, while important, do not necessarily capture how individuals perceive these factors as barriers to achieving adherence. Furthermore, even instruments specifically targeting perceived barriers, as the Perceived Social Support (PSS-HIV) [23] and the Perceived HIV Self-Management Scale (PHIVSMS) [24], address isolated barriers rather than the complex, interacting set of challenges that hinder ART adherence. As such, the development and availability of multidimensional instruments to assess perceived barriers to achieving ART adherence is warranted [25].

The Perceived Barriers to Antiretroviral Therapy Adherence (in Portuguese, Percepção de Dificuldades com o Tratamento Antirretroviral—PEDIA) scale is an instrument in Brazilian Portuguese comprising 18 statements that describe challenges of living with HIV and barriers to taking ART medication. This instrument was developed through an initial qualitative analysis of 3 open-ended questions on the aspects that facilitate and hinder daily ART adherence which were answered by 598 people living with HIV across all Brazilian regions. Subsequently, 47 statements were elaborated and a content validity was performed, eliminating 7 items [16]. The final version of the scale was proposed after its evaluation in a sample of people living with HIV receiving health care in Belo Horizonte, Brazil [26].

As hypothesized, the PEDIA scale may be able to predict ART nonadherence, and thus, provide an opportunity to intervene before the therapy fails. In addition, it can aid our understanding of the specific barriers faced by MSM and TGNB persons living with HIV, however an assessment of the instrument’s validity in this population is lacking. In this study, we sought to evaluate the psychometric properties of the PEDIA scale among MSM and TGNB persons in Brazil.

## Methods

### Study Design

Using a cross-sectional study design, we evaluated the factor structure, construct validity, and internal reliability of the PEDIA scale using 2 convenience samples recruited through internet-based surveys in Brazil.

### Study Population

A total of 2 independent samples of Brazilian MSM and TGNB persons were recruited through location-based dating apps and social media. Sample 2020 was recruited between February 20, 2020, and March 29, 2020 through Hornet [Queer Networks Inc.], where users received an inbox invite to participate in the research [27]. Sample 2021 was recruited between May 14, 2021, and January 29, 2022 using 3 different location-based dating apps (Hornet, Grindr [Grindr LLC], and Scruff [Perry Street Software]) and social media (Facebook and Instagram [Meta Platforms]) [28]. Recruitment strategies varied by app: direct inbox messaging was used for Hornet, whereas banners were used on Grindr and Scruff. Boosted posts were used on Facebook and Instagram. Individuals were not compensated for their participation as per Brazilian research regulations.

Adult (age ≥18 y) participants who provided electronic informed consent were directed to the study’s questionnaires. Participants who (1) identified themselves as cisgender women, (2) reported living abroad, (3) incorrectly answered any of the attention questions (only applicable to Sample 2020) [29], or (4) abandoned the questionnaire before reaching the last question were excluded. Furthermore, in line with the study’s objective to evaluate an instrument addressing barriers to sustaining ART adherence, participants who reported their HIV status as negative or unknown were excluded. Duplicate responses were identified and removed based on the IP address, with only the first instance retained for analysis. To protect respondent privacy, a masked IP was generated, used for duplicate removal and then deleted.

### Recruitment Apps

Grindr, Hornet, and Scruff are geosocial networking (GSN) apps designed for dating and sexual encounters mostly used by MSM. According to the Google play store, as of July 2024, Grindr and Hornet had each surpassed 10 million downloads and Scruff, 5 millions in Brazil alone. These apps are quite popular among MSM and recruitment based on them has been previously used in other cross-sectional studies in Brazil [27,28,30-35]. Instagram and Facebook are social media apps widely used in Brazil. Although these figures vary over time, as of June 2024, 29.6% of Brazilians actively use Instagram, whereas 33% use Facebook [36].

### Study Instruments

Questionnaires were administered via Alchemer and consisted of about 100 questions, though the actual number of questions was dependent on the participant’s responses given conditional logic. Participants were able to change and review any of their answers and always had the possibility to choose a nonresponse option, usually labeled as “Prefer not to answer.” Usability and technical functionality of questionnaires were reviewed in different devices and operating systems before launching the study.

The questionnaires had 3 main sections. Section 1 included sociodemographic characteristics of the participants (age, gender, sexual orientation, race, education, income, and state of residency). Section 2 addressed the use of ART, and if in use, the level of ART adherence. Section 3 focused on possible barriers to achieve ART adherence, such as HIV stigma, people-reported barriers, alcohol use, drug use, among others.

The WebAd-Q is an instrument developed and validated in Brazil to assess ART adherence. The validity and internal consistency were evaluated in a sample of 74 adults taking ART for at least 3 months. Construct validity was supported by high correlations between responses to the WebAd-Q items and 5 different measures of ART adherence. Test-retest measures indicated a Kappa coefficient of 0.77 [37]. The WebAd-Q instrument consists of 3 questions inquiring on not following scheduled hours for ART intake, missing ART doses, and incorrectly taking more or less pills than prescribed. For all questions, the recall period was 7 days and the response options included “Yes,” “No,” or “I don’t remember.” As per instrument’s instructions, a participant was deemed adherent if they answered “No” to all 3 questions. Following the three WebAd-Q questions, the participants were prompted with a visual analogue scale (VAS) item: “please mark below the value that corresponds to how much of your antiretroviral medication you took in the past 30 days” that varied from 0% to 100%. A dichotomous variable, denominated “adherence to ART based on VAS dummy,” was created by categorizing <100% as nonadherence and 100% as adherence [38,39]. In sum, the 3 measures of ART adherence were the WebAd-Q instrument, the 0%‐100% VAS, and the “Yes/No” adherence based on VAS.

The PEDIA scale is an instrument developed in Brazil to assess various barriers to achieve ART adherence as reported by people living with HIV. Initial validity of the PEDIA scale was assessed in a study of 415 adults accompanied in 3 health care facilities in Brazil, with results showing good reliability (McDonald’s omega of 0.97) and construct validity (one point increase in PEDIA’s score increases the odds of nonadherence to ART in 12%) [26]. PEDIA’s 18 statements should be answered on a 3-point Likert scale of agreement: “disagree” (coded as 1), “neither agree nor disagree” (coded as 2), and “agree” (coded as 3). Items 5 and 10 were reverse coded so that all statements reflect barriers and challenges of living with HIV and of daily medication. An overall score of the scale is given by the sum of each item’s response, with higher scores indicative of more perceived barriers (score range 18‐54). PEDIA captures 3 different dimensions, labelled as “patient’s fears and feelings,” “cognitive and routine problems,” and “medication and health concerns” (see Table 1). For each dimension, the corresponding score is also calculated by summing the respective items, the score range of each dimension is as follows: “patient’s fears and feelings,” 8 items, score range 8‐24, “cognitive and routine problems,” 4 items, score range 4‐12, and “medication and health concerns,” 6 items, score range 6‐18.

**Table 1.** The 18 items of the perceived barriers to antiretroviral therapy adherence (PEDIA) scale by dimension (adapted from the original paper [26]).

Item number	Item content
Patient’s fears and feelings	
1	The main problem of living with HIV is the stigma around it.
2	I am afraid to be identified as living with HIV when I go to the health care facility to get my HIV meds refill.
3	It frustrates me to think that I need to take the HIV meds in order to be alive.
7	I do not like to take my HIV meds around others.
11	It is tiresome to take my HIV meds every day.
14	It is difficult to tell people that I am living with HIV.
15	I am worried about the HIV meds stopping to work in the future.
17	It bothers me that I have to get my HIV meds refill in the health care facility’s pharmacy.
Cognitive and routine problems	
6	It is difficult to take my HIV meds at home.
8	Sometimes I forget to take my HIV meds because I get distracted.
9	It is difficult to take my HIV meds at work.
18	It is harder to keep track of my HIV meds on weekends.
Medication and health concerns	
4	Sometimes I skip taking my HIV meds because I want to avoid side effects.
5	Despite my HIV status, I live a normal life.
10	I believe that my HIV meds make me healthy.
12	I find it difficult to swallow the pills.
13	When I feel depressed I do not want to take my HIV meds.
16	It is hard to get used to the side effects.

### Statistical Analysis

We used a split-sample approach, using separate samples for exploratory factor analysis (EFA) and confirmatory factor analysis (CFA). All EFAs were conducted using Sample 2020, and all CFAs were conducted using Sample 2021.

#### Sample 2020: Exploratory Factor Analysis

First, we calculated the Kaiser-Meyer-Olkin (KMO) measure of sampling adequacy and performed the Bartlett test of sphericity to explore the presence of latent factors and the suitability of the data for EFA [40]. The KMO measure was deemed acceptable if greater than 0.6 and the significance level for the Bartlett test of sphericity was 0.05.

Then, we proceeded to implement an EFA (EFA 1) with polychoric correlation matrix and robust diagonally weighted least squares as the extraction method, given the 3-points Likert scale response format [41]. We first considered the originally proposed 3-factor structure, using parallel analysis (PA) with minimum rank factor analysis to determine the optimal number of factors [42] and robust promin rotation [43]. Based on the PA result, we reran our EFA (EFA 2) with the same specifications aforementioned and the optimal number of factors given by the PA. Items with standardized factor loadings below 0.4 were removed from subsequent analyses, aiming a more meaningful and robust factor structure [44,45]. While theoretical relevance was also considered during item removal, the primary criterion was the 0.4 factor loading threshold. Low loadings indicate that the item does not strongly represent the factor, and retaining such items can add noise and reduce the clarity of the factor structure [46].

#### Sample 2021: Confirmatory Factor Analysis

Akin to the initial steps undertaken for Sample 2020, we first calculated the KMO measure of sampling adequacy and performed Bartlett’s test of sphericity to assess the presence of latent factors and the suitability of the data for CFA. We used the optimal number of factors with the remaining items from our second EFA to run this CFA. Our CFA was performed using a polychoric correlation matrix and weighted least squares estimator with a diagonal weight matrix and mean- and variance-adjusted *χ*^2^ test statistic (WLSMV), robust standard errors and delta parametrization [41]. Similarly to our EFA results, we removed items with standardized loadings below 0.4 [44,45].

In addition, modification indices were used to identify changes that could improve model fit such as items that were loading on more than 1 factor, or items heavily correlated with other items, among others. Theoretically plausible modifications were discussed by the study team. Finally, a second CFA was performed on the final version of the scale using a polychoric correlation matrix and WLSMV estimator, robust standard errors, and delta parametrization [41].

Since the optimal number of factors given by the PA was different from that of the original PEDIA scale, we performed a CFA on the original PEDIA scale with all the original items grouped into 3 factors, as described previously. We sought to show the difference in model fit and factor loadings for this version (see Table S1 in Multimedia Appendix 1).

Model fit was assessed using root mean square error of approximation (RMSEA), comparative fit index (CFI), Tucker-Lewis index (TLI), and standardized root mean square residual (SRMR). The *χ*^2^ statistic was not emphasized in our results due to its sensitivity to sample size and rejection of well-fitting models [47]. Model fitting was deemed acceptable if CFI≥0.95, TLI≥0.95, RMSEA≤0.06, and SRMR≤0.08 [48].

Construct validity was verified by standardized factor loadings above 0.5 and correlation assessment of the final version of the PEDIA scale with the 3 previously described measures of ART adherence. A negative correlation between perceived barriers as measured by PEDIA and ART adherence was taken as evidence of construct validity [49]. Spearman correlation coefficient was used for continuous variables, whereas point-biserial correlation was employed for categorical variables.

Internal consistency (reliability) was assessed using McDonald’s omega, which is considered an appropriate measure when handling ordinal variables (polychoric correlation matrices) and non-normally distributed variables [46]. Convergent factorial validity was evaluated based on the average variance extracted (AVE), which indicates convergence when √AVE ≥0.7 [50]. Finally, discriminating factorial validity was assessed by testing the statistical difference between the AVE of each factor and the square of its correlations with others [50]. If the difference was statistically not significant at 5%, then discriminating factorial validity was endorsed [51].

**Figure 1.**
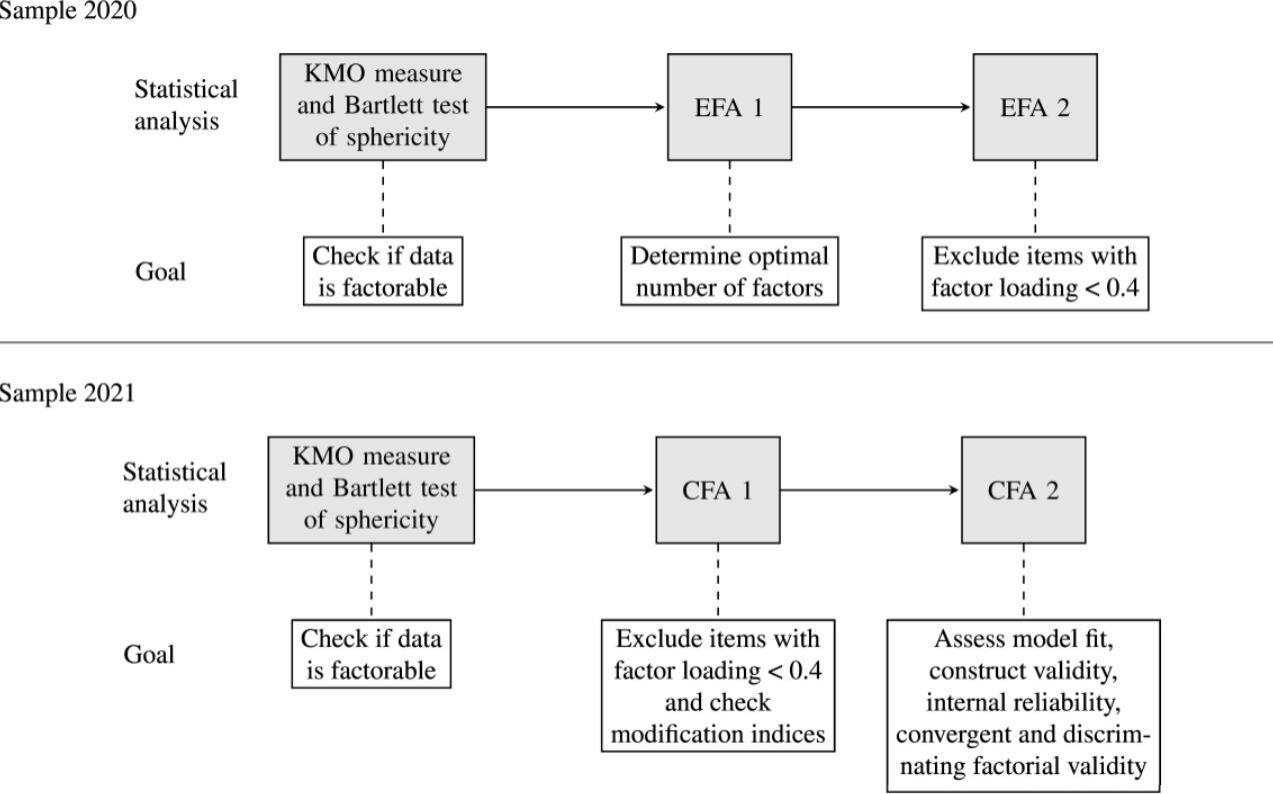
Flowchart of all statistical analyses performed to define the final version of perceived barriers to antiretroviral therapy adherence (PEDIA) and their goals by sample. CFA: confirmatory factor analysis; EFA: exploratory factor analysis; KMO: Kaiser-Meyer-Olkin.

Figure 1 shows the statistical analyses and their goals as performed in each sample. EFAs were conducted in Factor 12.04.05 Lorenzo-Seva and Ferrando [52] and CFAs in MPLUS 8.8 Muthén and Muthén [53]. Correlations were calculated in R (version 4.2.3; R Foundation for Statistical Computing) [54].

### Ethical Considerations

This study received approval from the human subject’s ethics committee at Instituto Nacional de Infectologia Evandro Chagas of Fundação Oswaldo Cruz. The study received the following ethics approvals: #2.531.022, issued on March 7, 2018, and #4.300.120, issued on September 25, 2020. All study participants provided electronic informed consent before survey initiation. To prevent duplicate responses, IP addresses were collected and subsequently discarded after data cleaning. No other personally identifiable information was collected. Individuals were not compensated for their participation as per Brazilian research regulations.

## Results

### Participant Characteristics

**Figure 2.**
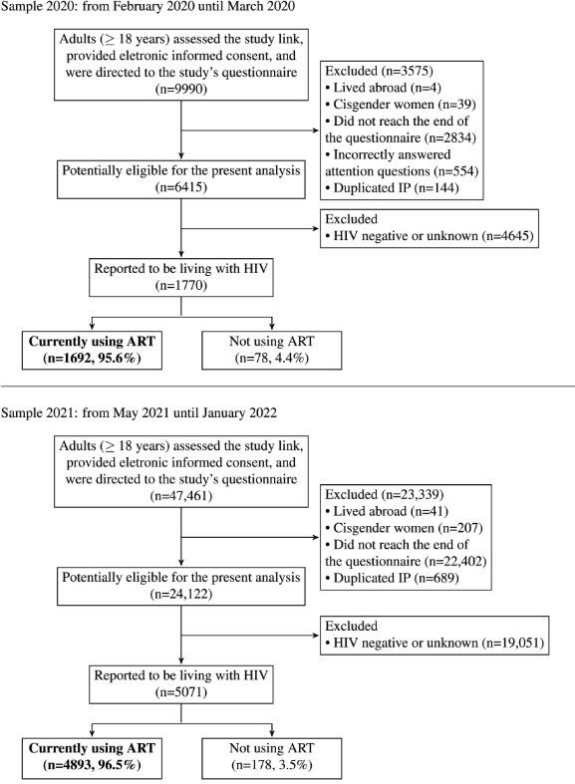
Flowchart of the study population and exclusion criteria by sample. ART: antiretroviral therapy.

Recruitment for Sample 2020 reached 9990 adults, out of which 3575 were excluded (see Figure 2 for reasons). Amidst those living with HIV (n=1770), 4.4% (n=78) reported not having initiated ART and 95.6% (n=1692) reported having initiated ART. Recruitment for Sample 2021 reached 47,641 adults, out of which 23,339 were excluded (see Figure 2 for reasons). Amidst those living with HIV (n=5071), 3.5% (n=178) reported not having initiated ART or chose not to answer this question whereas 96.5% (n=4893) reported having initiated ART. In total, Sample 2020 and Sample 2021 were composed, respectively, of 1692 and 4893 participants who were living with HIV and had initiated ART (Figure 2).

In Sample 2020 (see Table 2), median age was 36 years (IQR 30‐44 y), most participants were cisgender men (n=1665, 98.4%), self-identified as gay (n=1555, 91.9%) and White (n=1024, 60.5%), reported having a college degree or higher (n=1158, 68.4%), and were living in the Southeast region of Brazil (n=1386, 81.9%). A total of 971 (57.4%) were deemed adherent to ART based on the WebAd-Q. According to the VAS, 69.6% (n=1778) participants indicated 100% adherence to ART. Median PEDIA total score was 36 (IQR 32‐40), and breaking down by dimension, median scores were 17 (IQR 14‐19) for patient’s fears and feelings, 6 (IQR 4‐8) for cognitive and routine problems, and 11 (IQR 10‐12) for medication and health concerns. Sample 2021 exhibited a more diversified regional distribution (region of residency) when compared with Sample 2020 and some small *P* values emerged in demographic comparisons. All measures of ART adherence and PEDIAr scores were similar across samples (see Table 2).

**Table 2.** Characteristics of the 2 study samples.

Characteristics	Sample 2020 (n=1692)	Sample 2021 (n=4893)	*P* value
Age (years), median (IQR)	36 (30-44)	38 (31-45)	<.001
Age group (years), n (%)			.003
18‐24	90 (5.3)	241 (4.9)	
25‐29	283 (16.7)	651 (13.3)	
30‐39	667 (39.4)	1900 (38.8)	
40‐49	392 (23.2)	1317 (26.9)	
50‐59	223 (13.2)	679 (13.9)	
60+	37 (2.2)	105 (2.1)	
Gender, n (%)			.68
Cisgender man	1665 (98.4)	4800 (98.1)	
Trans man	2 (0.1)	5 (0.1)	
Trans woman	2 (0.1)	9 (0.2)	
Travesti	23 (1.4)	74 (1.5)	
Nonbinary	0 (0)	5 (0.1)	
Sexual orientation, n (%)			.003
Gay	1555 (91.9)	4344 (88.8)	
Bisexual	118 (7.0)	451 (9.2)	
Heterosexual	7 (0.4)	41 (0.8)	
Other^a^	12 (0.7)	57 (1.2)	
Race, n (%)			.003
Asian	20 (1.2)	43 (0.9)	
Black	173 (10.2)	577 (11.8)	
Pardo/mixed	419 (24.8)	1426 (29.1)	
Indigenous	18 (1.1)	52 (1.1)	
White	1024 (60.5)	2767 (56.6)	
Missing	38 (2.2)	28 (0.6)	
Schooling, n (%)			.08
High school or less	514 (30.4)	1646 (33.6)	
Undergraduate degree	650 (38.4)	1819 (37.2)	
Graduate degree	508 (30.0)	1416 (28.9)	
Missing	20 (1.2)	12 (0.2)	
Household income^b^, n (%)			.13
Low	457 (27)	1428 (29.2)	
Middle	776 (45.9)	2116 (43.2)	
High	459 (27.1)	1297 (26.5)	
Missing	0 (0)	52 (1.1)	
Region of residency, n (%)			<.001
North	10 (0.6)	120 (2.5)	
Northeast	38 (2.2)	431 (8.8)	
Central-west	55 (3.3)	330 (6.7)	
Southeast	1386 (81.9)	3317 (67.8)	
South	203 (12.0)	695 (14.2)	
Adherence to ART^c^-based on WebAd-Q^d^ , n (%)			>.99
No	721 (42.6)	2087 (42.7)	
Yes	971 (57.4)	2806 (57.3)	
Adherence to ART based on VAS^e^, median (IQR)	100 (98-100)	100 (98-100)	.24
Adherence to ART based on VAS dummy^f^, n (%)			.17
No	514 (30.4)	1398 (28.6)	
Yes	1178 (69.6)	3495 (71.4)	
PEDIA^g^ scale			
Total score, median (IQR)	36 (32-40)	36 (32-40)	.03
Fear and feelings, median (IQR)	17 (14-19)	17 (14-19)	.11
Cognitive and routines problems, median (IQR)	6 (4-8)	6 (4-8)	.50
Medication and health concerns, median (IQR)	11 (10-12)	11 (10-12)	.01

a “Other” includes responses of asexual, pansexual, and other.

b Household income was based on the ratio of household income relative to the number of minimum wages monthly: low ≤2, middle >2 and ≤ 6, and high >6.

c ART: antiretroviral therapy.

d Answered “no” to all 3 questions.

e VAS: visual analog scale.

f Dummy equals 1 if answered “100” on VAS, and 0 otherwise.

g PEDIA: perceived barriers to antiretroviral therapy adherence.

### Exploratory Factor Analysis

The KMO measure of sampling adequacy was 0.870 and the Bartlett test of sphericity *P* value was <.001, indicating that the data was factorable and that the EFA could be conducted. The first EFA (EFA 1) was performed using the original number of factors, that is, 3. The parallel analysis based on minimum rank factor analysis indicated the presence of 2 factors. Accordingly, the EFA was repeated (EFA 2) using 2 factors (see Table 3). Results showed that most items had standardized factor loadings ≥0.4 except for items 5, 10, and 16 and these items were thus removed from subsequent analyses (see Table 3).

**Table 3.** Standardized factor loadings for the perceived barriers to antiretroviral therapy adherence (PEDIA) scale by type of factory analysis used.^a^

		EFA 2		CFA 1		CFA 2	
Item number	Item content	F1	F2	F1	F2	F1	F2
1	The main problem of living with HIV is the stigma around it.	0.518	−0.232	0.363^b^	—^c^	—	—
2	I am afraid to be identified as living with HIV when I go to the health care facility to get my HIV meds refill.	0.918	−0.276	0.646^b^	—	—	—
3	It frustrates me to think that I need to take the HIV meds in order to be alive.	0.472	0.200	0.633	—	0.673	—
4	Sometimes I skip taking my HIV meds because I want to avoid side effects.	−0.067	0.793	—	0.647	—	0.648
5	Despite my HIV status, I live a normal life.	−0.229^d^	−0.366^d^	—	—	—	—
6	It is difficult to take my HIV meds at home.	−0.186	0.890	—	0.756	—	0.778
7	I do not like to take my HIV meds around others.	0.430	0.261	0.700	—	0.682	—
8	Sometimes I forget to take my HIV meds because I get distracted.	−0.265	0.781	—	0.600	—	0.638
9	It is difficult to take my HIV meds at work.	−0.096	0.850	—	0.818	—	0.829
10	I believe that my HIV meds make me healthy.	−0.117^d^	−0.312^d^	—	—	—	—
11	It is tiresome to take my HIV meds every day.	0.390	0.434	0.775	—	0.835	—
12	I find it difficult to swallow the pills.	0.136	0.459	—	0.597^b^	—	—
13	When I feel depressed I do not want to take my HIV meds.	0.046	0.740	—	0.705	—	0.680
14	It is difficult to tell people that I am living with HIV.	0.869	−0.233	0.646^b^	—	—	—
15	I am worried about the HIV meds stopping to work in the future.	0.518	0.049	0.491^b^	—	—	—
16	It is hard to get used to the side effects.	0.161^d^	0.397^d^	—	—	—	—
17	It bothers me that I have to get my HIV meds refill in the health care facility’s pharmacy.	0.739	−0.039	0.699	—	0.606	—
18	It is harder to keep track of my HIV meds on weekends.	0.022	0.601	—	0.685	—	0.675

a Two types of analysis were conducted: exploratory factor analysis (EFA) and confirmatory factor analysis (CFA). All analyses used a 2-factor structure. EFA 2 was performed using Sample 2020. CFAs 1 and 2 were performed using Sample 2021.

b Item removal in CFA 1 was based on modification indices and theoretical relevance.

c Not applicable.

d Item removal in EFA 2 was primarily determined by standardized factor loadings below 0.4, supplemented by theoretical considerations.

### Confirmatory Factor Analysis

Fit indices for the 2-factor model of the PEDIA scale with 15 items were poor (RMSEA=0.08, 90% CI 0.081‐0.086; CFI=0.89; TLI=0.87; and SRMR=0.08). Items 1 (“The main problem of living with HIV is the stigma around it”) and 14 (“It is difficult to tell people that I am living with HIV”) do not specifically relate to the challenges of treatment. Items 12 (“I find it difficult to swallow the pills”) and 15 (“I am worried about the HIV meds stopping to work in the future”) tackle an issue that is not as relevant nowadays given the large advancements in the ART medication. In addition, item 1 did not load above 0.4, and items 12 and 15 were the lowest factor loadings in their corresponding factors. The team decided that these four items should be excluded (see Table 3).

The modification indices were then explored and discussed by the study team. Results showed that item 2 (“I am afraid to be identified as living with HIV when I go to the health care facility to get my HIV meds refill”) was strongly correlated with items 11, 14 and especially 17, suggesting that its content was overlapping with the aforementioned items. The idea proposed in item 2 (“I am afraid to be identified as living with HIV when I go to the health care facility to get my HIV meds refill”) also seemed already encompassed in item 17 (“It bothers me that I have to get my HIV meds refill in the health care facility’s pharmacy”). The team then decided to exclude item 2 (see Table 3).

**Figure 3.**
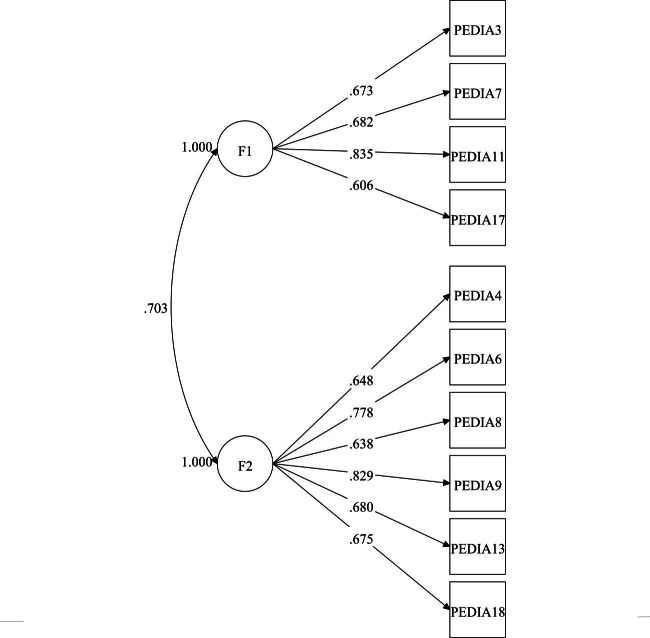
Standardized factor loadings for the reduced version of the perceived barriers to antiretroviral therapy adherence (PEDIAr) scale by factor and the correlation between the factors. F1: factor 1, labelled as "psychological aspects"; F2: factor 2, labelled as "practical aspects"; PEDIA: perceived barriers to antiretroviral therapy adherence.

Subsequent to the exclusion of items, the reduced version of the PEDIA scale, henceforth referred to as PEDIAr, retained 10 items with a 2-factor structure. Factor 1, labelled “psychological aspects,” encompassed items 3, 7, 11, and 17, whereas factor 2, labelled “practical aspects,” grouped items 4, 6, 8, 9, 13, and 18 (see Table 4). PEDIAr showed an acceptable fit (RMSEA=0.07, 90% CI 0.067‐0.075; CFI=0.95; TLI=0.94; SRMR=0.05). Construct validity was also supported by factor loadings well above 0.5 (see Figure 3). Internal consistency was verified by McDonald omega: 0.795 for factor 1, and 0.859 for factor 2. Both factors exhibited convergent factorial validity, as evidenced by their √AVE values of 0.704 and 0.711 for factor 1 and 2, respectively. In addition, discriminating factorial validity was supported, with no significant differences between the AVE of each factor and the square of its correlations with others: 0.001 (*P*=.94) for factor 1 and 0.009 (*P*=.55) for factor 2.

**Table 4.** The 10 items of the reduced version of the perceived barriers to antiretroviral therapy adherence scale (PEDIAr) by factor.

Item number	Item content
Psychological aspects	
3	It frustrates me to think that I need to take the HIV meds in order to be alive.
7	I do not like to take my HIV meds around others.
11	It is tiresome to take my HIV meds every day.
17	It bothers me that I have to get my HIV meds refill in the health care facility’s pharmacy.
Practical aspects	
4	Sometimes I skip taking my HIV meds because I want to avoid side effects.
6	It is difficult to take my HIV meds at home.
8	Sometimes I forget to take my HIV meds because I get distracted.
9	It is difficult to take my HIV meds at work.
13	When I feel depressed I do not want to take my HIV meds.
18	It is harder to keep track of my HIV meds on weekends.

Complementarily to the factor loadings, the correlations between the 10-item PEDIAr scale and all measures of ART adherence were negative and with a relevant magnitude, especially between factor 2 (6 items, labeled “practical aspects”) and ART adherence measured by WebAd-Q and between the total score of the PEDIAr and ART adherence measured by WebAd-Q in both samples (see Table 5). It is also noteworthy that the correlations were quite similar in the 2 samples.

**Table 5.** Correlations of the reduced version of the perceived barriers to antiretroviral therapy adherence scale (PEDIAr)—by factor and total score—and antiretroviral therapy (ART) adherence measures.

	Type	Sample 2020 (95% CI)	Sample 2021 (95% CI)
PEDIAr Factor 1			
ART adherence WedAd-Q	Biserial	−0.187 (−0.232 to −0.140)	−0.183 (−0.210 to −0.156)
ART adherence VAS^a^	Spearman	−0.175 (−0.221 to −0.129)	−0.147 (−0.175 to −0.120)
ART adherence VAS dummy^b^	Biserial	−0.165 (−0.211 to −0.118)	−0.136 (−0.164 to −0.109)
PEDIAr Factor 2			
ART adherence WedAd-Q	Biserial	−0.517 (−0.551 to −0.482)	−0.496 (−0.517 to −0.475)
ART adherence VAS	Spearman	−0.442 (−0.461 to −0.382)	−0.395 (−0.418 to −0.371)
ART adherence VAS dummy	Biserial	−0.407 (−0.446 to −0.366)	−0.376 (−0.340 to −0.352)
PEDIAr (10-items)			
ART adherence WedAd-Q	Biserial	−0.432 (−0.470 to −0.392)	−0.408 (−0.431 to −0.384)
ART adherence VAS	Spearman	−0.355 (−0.396 to −0.313)	−0.315 (−0.340 to −0.289)
ART adherence VAS dummy	Biserial	−0.349 (−0.391 to −0.307)	−0.308 (−0.333 to −0.282)

a VAS: visual analog scale.

b ART adherence VAS dummy=1, if the participant marked 100 on VAS, 0 otherwise.

## Discussion

### Principal Findings

As described previously, the PEDIA scale was first assessed among 415 people living with HIV (n=336, 81% men and n=79, 19% women; mean age 35, SD 10.45 years) using ART from 3 health care facilities in the city of Belo Horizonte, Brazil, who were recruited between September 2015 and October 2017. In this study, the original 3-factor structure of the PEDIA scale was not supported by the parallel analysis carried out in our EFA 1 and was further disconfirmed by its CFA fit indices that did not meet any of the conventionally used cutoffs (see Table S1 in Multimedia Appendix 1). Therefore, for our population that was predominantly composed of cisgender MSM (n=4795, 98%, mean age 39, SD 9.84 years), we proposed a reduced version, the PEDIAr scale, for which we have provided evidence of construct validity, convergent and discriminating factorial validity, and a well-fitted 2-factor structure. As recommended [46,51], this proposed refinement of the PEDIA scale was achieved based on both item-item and item-total correlations, along with theoretical relevance. Accordingly, we believe the PEDIAr scale retains PEDIA’s original aim to assess perceived barriers to adherence from the perspective of the person, that is, to assess which factors hinder daily adherence to ART.

In comparison with the original structure, some items initially assigned to the dimensions representing “cognitive and routine problems” (4 items) and “medication and health concerns” (6 items) were merged into a single dimension “practical aspects” with 6 items (items 5, 10, 12, and 16 of the original scale were excluded), whereas the dimension “patient’s fears and feelings” remained as one of the factors, renamed “psychological aspects,” with 4 items (items 1, 2, 14, and 15 were excluded). PEDIAr presented good fitness measures (TLI, CFI, and SRMR), good internal consistency provided by the McDonald’s omega, construct validity through high factor loadings, and convergent and discriminating factorial validity assessed by the AVE. Our results suggest a restructuring of the original 3-factor model into 2: one focusing on subjective barriers to daily medication adherence, including thoughts and feelings (denominated “psychological aspects”), and another focusing on the objective challenges to achieving daily adherence, including habit formation and normal disruptions to daily routines, hence the denomination “practical aspects.”

Some items of PEDIAr tangentially refer to internalized HIV-related stigma and depressive symptoms, which are factors that intertwine with perceived barriers to sustain ART adherence. Item 9 (“It is difficult to take my HIV meds at work”) touches on the stigma faced by people living with HIV, that is, taking antiretroviral drugs in public or alongside peers poses a threat to the person’s well-being and sense of worth [55]. Item 13 (“When I feel depressed I do not want to take my HIV meds”) alludes to the intricate relationship between depression and ART adherence. A meta-analysis across 29 studies found that the treatment of depression and psychological distress improved ART adherence [56]. Recent longitudinal evidence from Uganda (n=143) suggests a bidirectional relationship between depression and ART adherence [57]. Furthermore, other intersecting stigmas, such as internalized homonegativity, might play a role deterring ART adherence through the aforementioned factors. There is a large body of literature showing that internalized homonegativity is positively associated with depressive symptoms [58-60]. Similarly, a meta-analysis including 176 studies published between 2000 and 2021 found a positive association between internalized homonegativity and various forms of HIV-related stigma, including internalized HIV stigma [61]. When people living with HIV’s ability to adhere to ART is compromised, PEDIAr overall scores would likely increase. However, further research is needed to specifically examine the relationship between internalized homonegativity and PEDIAr scores.

Our results provide further evidence on PEDIAr’s construct validity based on the correlations found between the scale and ART adherence [49]. Not only did the correlations show a relevant magnitude and expected direction of effect (ie, higher PEDIAr scores correlate with poor adherence), but they were consistent through varied measures of ART adherence. Furthermore, the results were similar in both samples, which corroborates to the reliability of the scale [51]. In this regard, it is important to highlight that some of the items excluded in our analysis did not tackle issues directly related to ART adherence such as items 1 (“The main problem of living with HIV is the stigma around it”), 5 (“Despite my HIV status, I live a normal life”), and 14 (“It is difficult to tell people that I am living with HIV”), while others were excluded because they referred to issues that were more prevalent with older, less efficient and more toxic medications, such as items 4 (“Sometimes I skip taking my HIV meds because I want to avoid side effects”), 12 (“I find it difficult to swallow the pills”), 15 (“I am worried about the HIV meds stopping to work in the future”), and 16 (“It is hard to get used to the side effects”), which are likely less relevant nowadays [62]. Taken together, these findings suggest that PEDIAr may be more specific in its assessment of current barriers to ART adherence.

In comparison with other existing instruments, most assess specific constructs derived from qualitative research with people living with HIV that have been shown to correlate with ART adherence [25]. Other instruments which assess perceived barriers per se, meaning the perception of factors as barriers to adherence, do it as unidimensional scales and this can hinder relevant information about the interplay among them [63]. In this instance, a major advantage of the original PEDIA scale is its multidimensionality, which was retained in PEDIAr. Furthermore, PEDIAr was consistently correlated with ART adherence, an essential attribute to be used as an insightful measure of a person’s perception of barriers to sustain ART adherence [26]. In terms of clinical practice, PEDIAr offers the advantage of brevity, comprising only 10 items, and may be self-administered by people living with HIV during routine visits, reducing time burden for both people seeking care and health care providers. While further research may be needed to explore its application within other populations, our study offers compelling evidence of PEDIAr’s applicability, especially among MSM, suggesting its value as a tool for routine clinical assessments.

Our study has some limitations that should be acknowledged. First, our sampling strategy relied on GSN apps and social media and thus results may not be generalizable to all Brazilian gay, bisexual and other MSM or, to an even greater extent, to other TGNB persons. Adding to the sampling limitations, both samples were primarily composed of individuals residing in the Southeast region of Brazil, the most populous and wealthiest region, potentially limiting the generalizability of our findings to other regions of the country. Nonetheless, given the challenges of reaching MSM and TGNB populations, especially in a nationwide context, the HIV prevalence in both samples was consistent with national studies targeting similar population groups, suggesting a degree of representativeness within the inherent limitations of internet-based sampling. In Sample 2020, HIV prevalence was estimated at 26.4% (n=6415), slightly higher than the average reported for Brazil in a respondent-driven sampling carried out in 12 cities in 2016 (n=3952, 18.4%) [64]. However, Sample 2020 was largely composed of participants from the southeast region, which exhibited a higher prevalence in the aforementioned study, especially in Rio de Janeiro (n=256, 15.3%) and São Paulo (n=341, 24.8%). In contrast, Sample 2021 was more diversified, capturing participants from other regions of Brazil. As a consequence, HIV prevalence found in Sample 2021 (n=24,122, 21.0%) was similar to the Brazilian average. Another limitation of our study is the possibility that participants from Sample 2020 were also included in Sample 2021 (meaning that they answered both surveys), which would violate the assumption of independence of our samples. However, we assessed the extent of this overlap based on the IP address and found it to be minimal: only 12 overlapping responses were identified, representing less than 1% of the total number of participants. Given the small magnitude of this overlap and that our split-sample approach does not rely on strict independence between the two samples, we did not remove these duplicates from the analysis. In addition, both of our instruments (PEDIA scale and ART adherence) were self-reported, which could be subject to recall and social desirability bias. However, existing evidence indicates that these self-reported measures are reliable [26,37].

### Conclusions

Perceived barriers to adherence to ART can help address an important challenge faced by ~30% to 40% of people living with HIV across different settings: nonadherence to therapy. This study evaluated the original PEDIA scale in 2 large samples of MSM and TGNB persons living with HIV and validated a reduced version of the instrument, the PEDIAr scale. PEDIAr offers a 2-factor structure that distinguishes between psychological and practical aspects influencing adherence. This distinction provides valuable insights into designing interventions to tackle specific challenges faced by people living with HIV. Our results also stress PEDIAr’s applicability, particularly among cisgender MSM, and its brevity and ease of administration make it accessible to diverse populations. Furthermore, shorter instruments not only reduce the participant’s burden, but may also increase retention. By identifying individuals struggling with adherence, PEDIAr can pave the way for personalized care and interventions, ultimately improving health-related outcomes. Future studies should explore PEDIAr’s performance in other populations and the relationship between barriers to adherence and ART adherence across different durations of ART use.

## Supplementary material

10.2196/67005 Multimedia Appendix 1 Standardized factor loadings of the original 3-factor perceived barriers to antiretroviral therapy adherence (PEDIA) scale using Sample 2021 data, and the reduced version of the PEDIA scale's final 10 items in Brazilian Portuguese, grouped by factor.

## Abbreviations

ART: antiretroviral therapy
AVE: average variance extracted
CFA: confirmatory factor analysis
CFI: comparative fit index
EFA: exploratory factor analysis
GSN: geosocial networking
KMO: Kaiser-Meyer-Olkin
MSM: men who have sex with men
PA: parallel analysis
PEDIA: perceived barriers to antiretroviral therapy adherence scale
PEDIAr: reduced version of the perceived barriers to antiretroviral therapy adherence scale
PHIVSMS: Perceived HIV Self-Management Scale
PSS-HIV: Perceived Social Support in HIV
RMSEA: root mean square error of approximation
SRMR: standardized root mean square residual
TGNB: transgender and nonbinary
TLI: Tucker-Lewis Index
VAS: visual analog scale
WLSMV: weighted least squares estimator with a diagonal weight matrix and mean- and variance-adjusted χ2 test statistic
