# Supplementary material for: Validation of the Perceived Barriers to Antiretroviral Therapy Adherence (PEDIA) Scale Among Gay, Bisexual, and Other Men Who Have Sex With Men and Transgender and Nonbinary Persons: Cross-Sectional Study
Source: JMIR Public Health Surveill. 2025 Jun 27;11:e67005. doi: 10.2196/67005 (PMC12256708; doi:10.2196/67005)
Supplement: Multimedia Appendix 1 [file publichealth-v11-e67005-s001.docx]

**Supplementary material**

The standardized factor loadings from the CFA for the original Perceived Barriers to Antiretroviral Therapy Adherence (PEDIA) scale with three factors using data from Sample 2021 are shown in Table S1. Goodness of fit measures were RMSEA = 0.072 (IC95: 0.069-0.074), CFI = 0.891, TLI = 0.874, and SRMR = 0.075.

Table S1: Standardized factor loadings for the original PEDIA scale with three factors

|  |  | **CFA** | | |
| --- | --- | --- | --- | --- |
| Item number | Item content | **Patients' fears and feelings** | **Cognitive and routine problems** | **Medication and health concerns** |
| 1 | The main problem of living with HIV is the stigma around it | 0.342 |  |  |
| 2 | I am afraid to be identified as HIV positive when I go to the healthcare facility to get my HIV meds refill | 0.633 |  |  |
| 3 | It frustrates me to think that I need to take the HIV meds in order to be alive | 0.639 |  |  |
| 4 | Sometimes I skip taking my HIV meds because I want to avoid side effects |  |  | 0.725 |
| 5 | Despite my HIV status, I live a normal life |  |  | 0.517 |
| 6 | It is difficult to take my HIV meds at home |  | 0.782 |  |
| 7 | I do not like to take my HIV meds around others | 0.695 |  |  |
| 8 | Sometimes I forget to take my HIV meds because I get distracted |  | 0.614 |  |
| 9 | It is difficult to take my HIV meds at work |  | 0.866 |  |
| 10 | I believe that my HIV meds make me healthy |  |  | 0.350 |
| 11 | It is tiresome to take my HIV meds everyday | 0.793 |  |  |
| 12 | I find it difficult to swallow the pills |  |  | 0.668 |
| 13 | When I feel depressed I do not want to take my HIV meds |  |  | 0.784 |
| 14 | It is difficult to tell people that I am HIV positive | 0.632 |  |  |
| 15 | I am worried about the HIV meds stopping to work in the future | 0.501 |  |  |
| 16 | It is hard to get used to the side effects |  |  | 0.614 |
| 17 | It bothers me that I have to get my HIV meds refill in the healthcare facility's pharmacy | 0.695 |  |  |
| 18 | It is harder to keep track of my HIV meds on weekends |  | 0.717 |  |

The reduced PEDIA (PEDIAr) consists of 10 items in Brazilian Portuguese, which are detailed in Table S2.

Table S2: PEDIAr’s final 10 items in Brazilian Portuguese by factor

| Número do item | Conteúdo do item |
| --- | --- |
| *Questões psíquicas* | |
| 3 | Não gosto da obrigação de ter que tomar o remédio para o HIV para poder viver |
| 7 | Não gosto de tomar o remédio para o HIV quando estou em público |
| 11 | É cansativo tomar os remédios para o HIV todos os dias |
| 17 | É um incômodo ter que buscar os remédios para o HIV na farmácia do serviço de saúde |
| *Questões práticas* | |
| 4 | Às vezes deixo de tomar os remédios para o HIV por medo de ter algum efeito desagradável |
| 6 | É difícil seguir os horários de tomar os remédios para o HIV quando estou em casa |
| 8 | Às vezes me esqueço de tomar o remédio para o HIV no horário por simples distração |
| 9 | É difícil seguir os horários de tomar os remédios para o HIV quando estou trabalhando |
| 13 | Quando me sinto deprimido não tenho vontade de tomar os remédios para o HIV |
| 18 | Nos finais de semana é mais difícil tomar os remédios |
